# Clinical Society of Maryland

**Published:** 1893-03

**Authors:** William T. Watson

**Affiliations:** Secretary; 1519 N. Broadway, Baltimore, Md.


### THE CLINICAL SOCIETY OF MARYLAND.

BALTIMORE, MD., December 16, 1892.

The 273d regular meeting of the Clinical Society was called to order by the President, DR. WM. E. MOSELEY.

DR. GEO. J. PRESTON read a paper on

### TRAUMATIC LESIONS OF THE SPINAL CORD, and showed many specimens illustrative of his subject. He considers the medical treatment of traumatic lesions as almost useless, and urges surgical treatment.

DR. J. W. CHAMBERS said that, in the past we have been in the habit of applying certain surgical principles to injuries of the head, which did not apply to injuries of the spinal cord. When a man has injury to his brain, no reasonable surgeon would hesitate to cut down and remove blood clots, etc. The same rule ought to apply to injuries of the spinal cord; we should cut down and remove spicula of bone, blood clots, etc., and put the wound in a condition of drainage. The anatomical conditions surrounding the spinal cord are not particularly favorable for the rapid absorption of blood clots. His experience, especially with experimental injuries upon dogs, leads him to believe the spinal cord is capable of more repair than is usually thought of. Certain localities of the spine in well nourished people are very difficult to operate upon, but convenience to the operator should not be a consideration in the case. So long as you do not increase the patient's danger, it is all right to operate.

While we cannot repair the parts of the cord which are destroyed primarily, we might prevent the injury which comes on secondarily from the pressure of the blood-clots and the irritation of the bony fragments. We might, in this way, avoid bed-sores, cystitis, and a number of other sequela that are as fatal, or more fatal, than the injury to the cord proper.

He had known a number of the cases reported by Dr. Preston. One of the cases, a young man, a doctor, was injured by diving in shallow water. He was dragged out by his friends, and in a little while consciousness returned, and he suffered intense pain in his arm and was almost totally unable to use his arm and his leg. Twenty-four hours after the injury he had no pain and no discomfort, and was perfectly intelligent. He remained in this condition about forty-eight hours. About six hours previous to his death it was noticed that he began to have a very high temperature—105 to 108.5, and two hours after death the temperature was 109 in the muscles of the back.

Another case was that of a young man diving in shallow water in almost the same spot as the first, and receiving exactly the same injury. He lived three days. No autopsy was held. The diagnosis was fracture of the fifth cervical vertebra. The first 24 hours he was apparently comfortable, joking with those around him, and enjoyed his food. The last 24 hours before death the temperature rose rapidly and was 108 an hour before he died. The temperature was 107 in the rectum two hours after death.

A third case was that of a man falling or jumping from a bridge into shallow water and brought into the hospital with symptoms of fracture of the upper portion of the cervical region. He did not have a temperature above 101. There was almost complete paralysis of legs and arms. He lived nearly three days.

Four of the cases related by Dr. Preston were fractured in about the same way, by diving into shallow water. The head, in these cases, did not give way, doubtless because it was cushioned, more or less, by the water. The fracture of the cervical vertebra was possibly due to excessive flexion. The most marked flexion would take place about the fifth cervical vertebra and this is the vertebra most frequently broken.

An interesting feature is the rise of temperature. The temperature of a man that had been hanged, when taken by Dr. Chambers fifteen minutes after death, was found to be 102. If the fracture is much above the fifth cervical vertebra, the man does not live long enough to let the temperature run up. The higher up the fracture, the more likely we are to have high temperature.

DR. J. M. T. FINNEY heartily endorsed what had been advocated by Drs. Preston and Chambers in regard to the surgical treatment of these cases. Very little harm has ever been done by operation, and in a number of cases a great deal of good has unquestionably resulted. Under the present system of antiseptic surgery, the question of possible harm being done can no longer be raised against surgical treatment. There is no class of patients that appeals to one's sympathies so much as those unfortunate ones of injuries to the spinal cord. Dr. Finney has seen a number of cases almost identical with those related by Dr. Preston. In one case where the fracture was about the 7th cervical vertebra, the man lived about six weeks. He had no elevation of temperature.

DR. PRESTON said that after injuries to the cord the sensory symptoms improved sooner and to a greater degree than the motor. The thing that kills the patient is often cystitis and bed-sores, and other trophic conditions, and operation would probably often allow enough repair to take place to prevent the trophic symptoms There is no doubt that in a great many of these cases the most important injury to the cord is certainly secondary, due to ensuing inflammation.

These cases are ones for legitimate experimentation; that is, cases of what may be called entire crush of the cord, for they are hopeless, as far as medical treatment is concerned.

The work of Abbe and others is certainly suggestive.

Whether we can ever do anything by joining nerve roots above and below the injury, as Abbe has tried to do, remains to be proven, but it is barely possible that that or similar operation may relieve the trophic influences, and may allow of sufficient conduction of impulses through the cord to bladder and rectum, etc., to keep the patients alive.

DR. EDWARD J. BERNSTEIN read a paper on

SKIASCOPY, OR OBJECTIVE OPTOMETRY.

DR. HARRY FRIEDENWALD considered this method of measuring the refraction of the eye as one of great advantage. It is the simplest of all the methods, and is one that can be easily demonstrated to students. The simplicity of it enables the general practitioner with instruments of great simplicity to measure the refraction easily and rapidly.

DR. FRIEDENWALD related

A CASE OF SYPHILIS OF THE EXTERNAL AUDITORY CANAL.

The patient was a colored man, 25 years of age. He had a chronic discharge of pus from his ear. The external auditory canal was filled with polypi. He made no improvement on the usual treatment for aural catarrh. It was found that the patient had recently had a chancre, and it was seen that the growth had the appearance of syphilitic papules. He was put upon antisyphilitic treatment, and within a very short time the polypi had entirely disappeared, leaving a clean auditory canal and a normal drum head. Syphiiis may affect the internal ear or middle ear, but it rarely affects the auditory canal, and this is evidently a rare case.

DR. J. H. BRANHAM related a case of

DRAINAGE OF A TUBERLOUS ABSCESS OF THE LUNG.

The patient has had phthisis for over a year. His lungs were in good condition excepting the middle lobe of the right lung, in which there appeared to be an abscess in the anterior part. The patient was failing rapidly, and suffering from a harrassing cough. The abscess was cut down upon about a week ago, and found to contain a teacupful of pus. The sixth rib was resected over the abscess, so as to allow of recession in case of improvement. The patient, since the operation, has been tolerably well; has some fever. The cavity has been washed out twice a day with antiseptic solutions. The abscess is filling up, and Dr. Branham is hopeful that it will heal entirely.

DR. CHAMBERS asked how much lung had to be cut through before the abscess was reached, and if there was much hemorrhage in the portion of the lung traversed in reaching the abscess.

DR. BRANHAM replied that the anterior wall of the abscess consisted of lung tissue thoroughly infiltrated with tubercle, and there were strong and complete adhesions between the lung and the parietal pleura. The abscess was about half an inch from the surface, and the tissue broke down readily with the handle of the scalpel and the finger nail. There was practically no hemorrhage.

DR. S. K. MERRICK told of a "Peculiar Case of Nasal Reflex." The patient came to Dr. Merrick's office on crutches. He had been under treatment for about six weeks for a pain in his right hip. He had a slight hypertrophy of the inferior turbinated bone in the nostril to which Dr. Merrick applied the galvano-cautery. In twenty-four hours the patient came back without crutches. He is now at work, and has perfect use of his limbs.

WILLIAM T. WATSON, Secretary,
1519 Broadway, Baltimore.

---

BALTIMORE, January 20, 1893.

The 275th regular meeting was called to order by the President, DR. WILLIAM E. MOSELEY.

DR. HARRY FRIEDENWALD exhibited a case of exophthalmos due to idiopathic hemorrhage into the orbit. One morning the patient, a robust negro man, arose feeling quite well. After washing, he felt a sudden pain in his eye, and found the eye protruding and sensitive. The eyeball was not at all injected, and the only reason for the exophthalmos that could be assigned was an orbital hemorrhage. The movements of the eye ball were very much restricted. During the next two days the pain was greatly diminished and the movements were increased. The vision of the affected eye has been greatly impaired since the hemorrhage. No blood has yet appeared under the conjunctiva. There are a very great number of cases reported of traumatic orbital hemorrhage with the same symptoms, but there are only a few cases of idiopathic hemorrhage on record.

The patient's history throws no light upon the cause of the hemorrhage.

Dr. Friedenwald remembered a similar case which he had seen in a colored man some years ago. The protrusion of the eyeball came on very suddenly, and could be ascribed to no other cause than orbital hemorrhage. These hemorrhages are by no means innocent, for the pressure which they exert upon the nerve of the eye is very prejudicial, and explains the rapid decrease in the patient's sight. In traumatic cases an accumulation of blood in the orbit is usually followed by total atrophy of the optic nerve.

DR. S. T. EARLE exhibited a Langdon Rectal Tube. This tube was introduced by Dr. Langdon, of the Miami Medical College, of Cincinnati, Ohio. It presents many advantages over the tube ordinarily in use. It is five feet in length, being intended to reach from the anus to the cecum. It is found extremely valuable in rectal alimentation, as the enemata may be carried up all the way into the large bowel, and is not followed by a desire for speedy evacuation as in the case where the nourishment is injected only into the rectum. The enemata by this means comes into contact with a larger absorbing surface. Another advantage of this tube is its resistance. It is $\frac{1}{2}$-inch tube with only $\frac{1}{8}$-inch opening. This prevents the kinking and bending upon itself which is so likely to take place in the ordinary rectal tube. The opening is at the end and not upon the side. This allows of an easy introduction, as by letting the enemata flow in gradually the bowel is distended in advance of the tube. In cases of fecal impaction this resistance and direct opening are of very decided advantage. This tube can be made to pass the fecal obstructions and throw the enemata above the obstruction. It is also valuable in the treatment of oxyuris vemicularis. It has been shown by Heller that the habitat of this worm is about the cecum and not about the rectum, as has been supposed. By this tube our medicaments can be thrown where they will do the most good.

DR. ROBERT W. JOHNSON related

### A DOZEN ACCIDENT CASES TREATED BY THE BLOOD-CLOT METHOD

—three gunshot wounds, two compound fractures of the skull, two compound fractures of patella, one compound fracture of tibia, one of compound dislocation of elbow, one punctured wound of knee-joint, one plastic amputation of breast, one strangulated testicle These cases were all eminently successful. They did not represent all of the series of cases treated by this method, but only the most successful ones. While the method is not universally successful, still, it may be often trusted, and when it fails, it does not leave the patient in a much worse position than had it not been tried.

DR. W. S. HALSTED congratulated Dr. Johnson upon his cases, and thought they were excellent illustrations of what could be done by the method.

There is a point that is not generally understood when the blood-clot method is spoken of. Schede, who advocated this method, made use of it to fill up dead spaces in bone to take the place of sponge, decalcified bone, cat gut and other foreign materials, and he contradicted the views of Bergman, who thought it was absolutely fatal to good results to allow blood to remain in the wounds. He did not, however, ascribe any beneficial antiseptic effects to the blood-clot. He did not know, at that time, of the experiments of Nutall, and of others since Nutall, which showed that the blood of some animals has the power to destroy germs, such as the typhoid germs, when inoculated with these germs. No one has yet succeeded in killing pyogenic organisms with human blood.

Perhaps the value of the method lies in the fact that we are no longer afraid of blood in a wound and so we do not feel anxious to obliterate dead spaces which are difficult to obliterate, and we do not have to prevent very minute arteries from bleeding. We have learned that blood is not very harmful in a wound, but we have not learned that it is a thing to be desired in a wound.

It is best to have a wound as dry as possible and to close it immediately, provided we can have it dry without constricting tissues with our ligatures and sutures. We have become sure that one of the most important things in technique is to avoid the strangulation of tissue. This is more important than the employment of antiseptics; more important than disinfecting the skin of the patient, and, perhaps, disinfecting our own hands, but not more important than disinfecting the ligatures.

DR. J. E. MICHAEL said that similar cases to those related by Dr. Johnson had been successfully treated by other methods. There can be no object in having a blood-clot except where there is a dead space to deal with. Where wounds can be made clean and closely approximated without constricting tissue, it seems reasonable to so treat them rather than to introduce an element that may be harmful.

DR. J. W. CHAMBERS.—We have about come to this conclusion: that a blood-clot is probably less injurious to tissue than a drainage-tube; that it does very little harm, whether it does good or not.

DR. RANDOLPH WINSLOW believed that wherever there are dead spaces, such as cavities in bone, which heal with difficulty by the process of granulation and cicatrization, the blood-clot affords a scaffolding by which the process of repairs are very much facilitated. He feels safer in making use of drainage where there are considerable cavities liable to be filled with fluids which may decompose, but in small cavities, or bone cavities, it is frequently desirable to have them filled with blood.

DR. A. FRIEDENWALD.—A blood-clot always forms after the enucleation of an eye. Before the period of antisepsis he always expected copious suppuration to follow enucleation, and it never failed. With antisepsis, he never had suppuration.

DR. JOHNSON said that, while the blood-clot will not destroy germs, and he had never intended to give the impression that it would, it will allow us to close up our wounds without too much interference. It is nature's method of filling up dead spaces and is what occurs in sub-cutaneous wounds and simple fractures. It enables us to do away with the drainage-tube to a great extent, and this is an extremely valuable addition to surgery. It makes each operation simpler. In endeavoring to check the hemorrhages of small oozing by sponge or gauze, or other means, we do injury to the tissues. We accomplish a good work by the avoidance of this injury.

W. T. WATSON, Secretary,
1519 N. Broadway.

---

BALTIMORE, February 3, 1893.

The 276th regular meeting was called to order by the President, DR. WM. E. MOSELEY.

DR. G. J. PRESTON exhibited a patient suffering with a peculiar nervous affection of his left arm. The patient—a man of 26—has done hard work all his life; has used his left hand more than his right. Last October he had a fall and struck on the shoulder and arm of the affected side, and the pathological condition developed soon after. The arm is very muscular, and there is no evidence of paralysis, no loss of sensation, and electric reflexes perfectly normal. The patient's hand assumes all sorts of queer positions, due to contracture of the muscles, and no sooner is one position reduced than another one immediately ensues, due to the contracture of a different group of muscles. These peculiar phenomena disappear entirely during sleep. Dr. Preston was inclined to believe that the condition was one of hysteria.

DR. WALTER B. PLATT read a paper upon

THE FACTOR OF AGE IN SURGICAL OPERATION.

His conclusions were based upon operations done by himself upon children and aged people. He has never seen any ill effects in children from confinement to bed for weeks and months, in hygienic surroundings. They stand confinement to bed and house better than any other age. In them ether anethesia is quickly induced, and consciousness quickly regained. He has never seen any bad effects on the circulatory system during or after the administration of anesthesia in children. The only case of shock which he had seen was in a case of osteo-myelitis of the femur where a great deal of suppuration had been going on for two or three years. A proportionate hemorrhage is probably better borne by a child than an adult. Children do not tolerate well an operation where the intestines are long exposed. Old persons having simply the ordinary senile changes, but being fairly healthy otherwise, can stand operation very well. Confinement to bed is an important matter in old people. If the injury compels them to lie flat on the back, hypostatic pneumonia may put an end to life. They should be watched carefully, and propped up if necessary.

DR. RANDOLPH WINSLOW reported "Some cases of severe compound fracture occurring in young boys."

DR. J. W. CHAMBERS said that the children stand the shock moderately well. He had recently operated upon a child in its second week for double harelip, with protrusion of the pre-maxillary. The child was under the anesthetic for three-fourths of an hour, but there was little or no shock. Children stand loss of blood fully as well as older people. They make blood very rapidly. It is very difficult to say how old people stand the shock for the reason that a large number of the old people operated upon are in a pathological condition independent of the condition for which they are operated upon. The tissues of old people heal about as well as those of young persons. If the patient's condition warrants an operation, and the heart and lungs and other organs are good, the question of age is hardly a factor.

DR. J. D. FARRAR read a paper upon

INTESTINAL ANTISEPSIS IN TYPHOID FEVER BY MEANS OF BISMUTH SUB-IODIDE AND SALOL.

He commended the plan in practice at the Cooper Hospital, Camden, N. J. He reported a series of 24 cases, with no deaths. The bismuth and salol were given in 5-grain doses, alternately, every three hours, night and day. No toxic symptoms from the drugs were noticed. This method of treatment is begun whenever diarrhea exists, and is continued throughout the disease. This treatment is thought to modify the severity, if it does not limit the duration of the disease. Tympanites is reduced, diarrhea controlled, and hemorrhage prevented.

DR. NORMENT said that one of the charts exhibited by Dr. Farrar showed the patient to have had a practically normal pulse, temperature and respiration, upon admission, and he thought that the case did not demonstrate so much the benefit of salol and sub-iodide of bismuth, as it did the benefit of convalescence. He thought that further trial was necessary before the utility of the method could be accepted.

W. T. WATSON, Secretary,
1519 N. Broadway.

